# Screening and identification of resistance related proteins from apple leaves inoculated with *Marssonina coronaria* (EII. & J. J. Davis)

**DOI:** 10.1186/1477-5956-12-7

**Published:** 2014-02-07

**Authors:** Miaomiao Li, Jianhua Xu, Zonghao Qiu, Juan Zhang, Fengwang Ma, Junke Zhang

**Affiliations:** 1 College of Horticulture, Northwest A & F University, Yangling, Shaanxi 712100, China; 2 State Key Laboratory of Crop Stress Biology in Arid Areas, Northwest A & F University, Yangling, Shaanxi 712100, China

**Keywords:** Apple, 2-DE, Comparative proteomics, *M. coronaria*, Quantitative real-time PCR

## Abstract

**Background:**

Apple, an invaluable fruit crop worldwide, is often prone to infection by pathogenic fungi. Identification of potentially resistance-conferring apple proteins is one of the most important aims for studying apple resistance mechanisms and promoting the development of disease-resistant apple strains. In order to find proteins which promote resistance to *Marssonina coronaria,* a deadly pathogen which has been related to premature apple maturation, proteomes from apple leaves inoculated with *M. coronaria* were characterized at 3 and 6 days post-inoculation by two dimensional electrophoresis (2-DE).

**Results:**

Overall, 59 differentially accumulated protein spots between inoculation and non-inoculation were successfully identified and aligned as 35 different proteins or protein families which involved in photosynthesis, amino acid metabolism, transport, energy metabolism, carbohydrate metabolism, binding, antioxidant, defense and stress. Quantitative real-time PCR (qRT-PCR) was also used to examine the change of some defense and stress related genes abundance under inoculated conditions.

**Conclusions:**

In a conclusion, different proteins in response to *Marssonina coronaria* were identified by proteomic analysis. Among of these proteins, there are some PR proteins, for example class III endo-chitinase, beta-1,3-glucanase and thaumatine-like protein, and some antioxidant related proteins including aldo/keto reductase AKR, ascorbate peroxidase and phi class glutathione S-transferase protein that were associated with disease resistance. The transcription levels of class III endo-chitinase (L13) and beta-1, 3-glucanase (L17) have a good relation with the abundance of the encoded protein’s accumulation, however, the mRNA abundance of thaumatine-like protein (L22) and ascorbate peroxidase (L28) are not correlated with their protein abundance of encoded protein. To elucidate the resistant mechanism, the data in the present study will promote us to investigate further the expression regulation of these target proteins.

## Background

The common apple (*Malus domestica*) has not only become one of the world's largest fruit crops, but has also served as an invaluable model organism for the study of commercial traits such as disease and pest resistance [1]. *Marssonina* apple blotch caused by the fungus *Diplocarpon mali* is a devastating defoliating disease which can infect apple tree leaves, twigs, and fruit during the growing season [2,3]. Infection usually occurs during periods of warm, damp weather conditions. Ascospores released from overwintered apothecia on leaves are considered the inoculum for primary infections, and conidia produced in acervuli are thought to be responsible for secondary infections during the apple growing season. This disease is first manifested by brown spots and dark green circular patches on the leaf upper surfaces in mid-Summer. As it progresses, those spots coalesce with each other and black pinhead-like asexual fruiting bodies develop on the affected tissue [4]. Severe infections often lead to premature defoliation, which weakens tree vigor and diminishes crop yield and quality. Infections usually occur in consecutive years and have become a serious problem at several apple production areas in China [5].

To solve this problem, a better understanding of the complex plant defense mechanisms against *Marssonina coronaria* infection is extremely important. The optimum conditions for *Marssonina coronaria* growth researched by Dong-Hyuk Lee which supply the method of bacteria culture in vitro for us [6]. Hua Zhao et al obtained the mode of *Marssonina coronaria* infection and infection progress using fluorescence and electron microscopy [7]. Lihua Yin et al reported that ‘Qinguan’ had signs of disease resistance, with a low incidence of *Marssonina coronaria* infection [8]. Q. Zhou et al described the characterization of defense-related genes in the ‘Qinguan’ apple in response to *Marssonina coronaria* by cDNA suppression subtractive hybridization analysis [9]. However, these studies were limited to epidemiology and gene level, not touch upon the level of protein.

The proteins are the final executors of most biological processes. Proteome analysis, which focuses on investigating accumulative changes and modifications of proteins, could lead to a more comprehensive understanding of biotic stresses in host plants [10]. Two-dimensional (2-D) gel electrophoresis has emerged as a powerful tool for the study of plant–stress responses to plant–herbivore and plant–pathogen interactions [11-13]. It has been successfully applied to analysis of proteomics in different plant [14-16]. Despite the promising results of these and other studies, no investigation has been made into the identification of potential disease resistance-related proteins in the *Marssonina* apple blotch.

This study seeks to obtain potentially resistant proteins, from the detached leaves from ‘Qinguan’ apples and analyze the proteome via two-dimensional (2-D) gel electrophoresis. Data from this study could be used to facilitate the development of plans resistant to the *Marssonina* apple blotch.

## Results

### Pathogenesis of *M. coronaria* on isolated apple leaves

**Figure 1:**
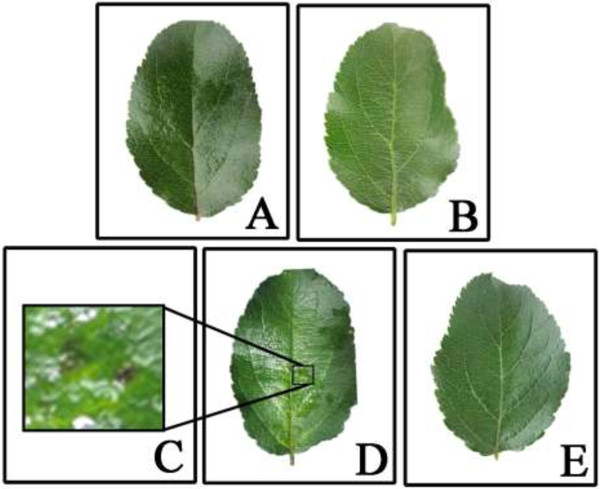
**Pathogensis of *M. coronaria *on isolated ‘Qinguan’ leaves after inoculation. A**. The isolated ‘Qinguan’ leaf inoculated with *M. coronaria* after 3 day. **B**. The isolated ‘Qinguan’ leaf non-inoculated after 3 day (treated with sterile water alone). **C**. Partially enlarged view of the lesion of isolated leaf 6 day post inoculation. **D**. The isolated ‘Qinguan’ leaf inoculated with *M. coronaria* after 6 day. **E**. The isolated ‘Qinguan’ leaf non-inoculated after 6 day (treated with sterile water alone).

Individual leaf symptoms of infection following inoculation with *M. coronaria* were observed and recorded. No visible symptoms including color or structure change had occurred at 3 day after inoculation compared to control. However, some small black lesions were observed at 6 day post inoculation, indicating successful inoculation and sufficient infection. Morphological changes observed during the infection are presented in Figure 1.

### Identification of *Marssonina coronaria*-responsive proteins by 2-DE and MS

**Figure 2:**
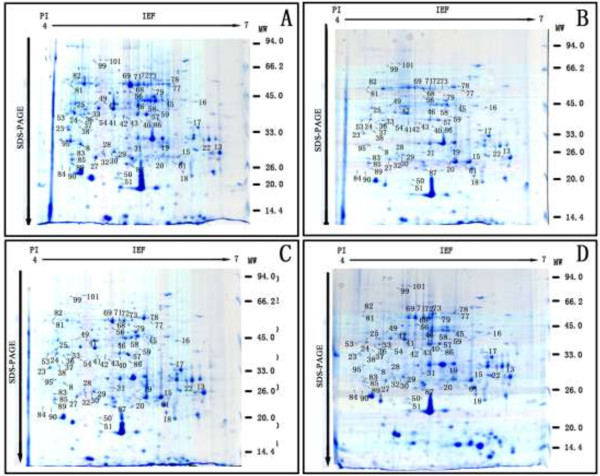
**Annotated gel images of ‘Qinguan’ leaf proteome.** 2-DE protein profiles of non-inoculated **(B, D)** and inoculated **(A, C)** leaves of ‘Qinguan’ in response to *Marssonina coronaria* infection. Significantly altered and identified spots are marked with corresponding spot number. The name of the protein is shown in Table 1. For the first dimension, 1000 *μg* of proteins were loaded on a 17 cm IPG strip with a linear gradient of pH 4-7, and 12% SDS-PAGE gels were used for the second dimension. The gels were stained by colloidal Coomassie brilliant blue G-250. M. molecular marker. **A**. Inoculated with *Marssonina coronaria* after 3 day. **B** Non-inoculated after 3 day (treated with sterile water alone). **C**. Inoculated with *Marssonina coronaria* after 6 day . **D**. Non-inoculated after 6 day (treated with sterile water alone).

In order to verify the change of different resistance-related proteins abundance between inoculated and non inoculated conditions, proteomic profiles of apple leaves were obtained via 2-D gel electrophoresis. A total of eighty-one protein spots showed significantly differential change in abundance between inoculated and control groups, see Figure 2. These spots were excised from the gel, and fifty-nine protein spots were identified successfully by peptide digestion followed by TOF/MS and proteomic peptide library identification via BLAST search and the results were listed out in Table 1. According the protein function, these proteins were aligned into thirty-five proteins or protein family.

**Table 1:** Proteins identified and analyzed by MALDI-TOF-TOF/MS

***Spot no***	***Protein name***	***Accession no.***	***Deduced apple genome ID***	***Protein MW/PI***	***Pep. count***	***Protein score***	***Relative protein content***
Photosynthesis proteins							
L8	ribulose-1,5-bisphosphate carboxylase	gi|2961315	MDP0000597996	53314.4/6.14	5	253	
L45	ribulose-1,5-bisphosphate carboxylase/oxygenase activase alpha 2	gi|78100212	MDP0000944409	46944.1/4.84	12	494	
L49	ribulose-1,5-bisphosphate carboxylase/oxygenase activase small protein isoform	gi|115334979	MDP0000321244	47952.1/7.57	10	326	
L50	Oxygen-evolving enhancer protein 2, chloroplastic; Short = OEE2;	gi|11134051	MDP0000361338	28674.3/6.6	4	231	
L51	ribulose 1,5-bisphosphate carboxylase	gi|4206530	MDP0000597996	49888.8/5.94	3	223	
L82	ribulose 1,5-bisphosphate carboxylase	gi|10945633	MDP0000597996	52133/6.18	8	154	
L85	ribulose-phosphate 4-epimerase	gi|225457361	MDP0000137234	30146/8.93	5	533	
L95	ribulose 1,5-bisphosphate carboxylase	gi|371928199	MDP0000597996	49673/6.04	20	498	
L15	Chlorophyll a-b binding protein 3C-like protein	gi|357497757	MDP0000784451	24884.4/5.53	4	133	
L19	putative chlorophyll a/b binding protein	gi|397789264	MDP0000875642	16135/7.82	6	262	
L61	Chlorophyll a-b binding protein 2, chloroplastic;	gi|158562858	MDP0000708928	3798.9/8.2	4	212	
L87	chlorophyll a-b binding protein 8, chloroplastic	gi|225436257	MDP0000866655	29511.2/7.85	2	192	
Amino acid metabolism related proteins							
L43	Serine-type peptidase	gi|357495999	MDP000065459	45945.4/6.79	9	267	
L54	precursor of carboxylase p-protein 1, glycine decarboxylase complex	gi|224088838	MDP0000588069	115985/6.51	11	407	
L58	putative plastidic glutamine synthetase	gi|26892040	MDP0000139493	47619.1/6.77	9	457	
Transpot related proteins							
L56	Transketolase, chloroplastic	gi|75140229	MDP0000142098	73346.7/5.47	6	20-6	
L81	alanine aminotransferase 3	gi|351724777	MDP0000815368	53789.3/5.52	11	470	
L99	PREDICTED: transketolase, chloroplastic-like	gi|356536526	MDP0000142098	80689.8/6.12	11	299	
L101	Transketolase	gi|357445031	MDP0000142098	80087.5/6.00	10	219	
Energy metabolism related proteins							
L23	PREDICTED: ferredoxin--NADP reductase, leaf isozyme, chloroplastic-like isoform 1	gi|356538289	MDP0000811918	40803.8/8.7	14	477	
L24	NAD dependent epimerase/dehydratase	gi|255559448	MDP000019372	43230.8/8.75	6	243	
Carbohydrate metabolism related proteins							
L27	PREDICTED: triosephosphate isomerase	gi|225449541	MDP0000152242	27396.2/6.34	5	373	
L33	alcohol dehydrogenase	gi|307135978	MDP0000239956	40657.6/8.53	3	138	
L36	cytosolic malate dehydrogenase	gi|78216493	MDP0000174740	35970.5/6.01	3	352	
L40	glyceraldehyde-3-phosphate dehydrogenase A	gi|381393060	MDP0000527995	43224.5/8.1	18	853	
L41	PREDICTED: fructose-1,6-bisphosphatase, cytosolic	gi|225460680	MDP0000251810	37554/6.03	7	332	
L42	glyceraldehyde-3-phosphate dehydrogenase B	gi|381393062	MDP0000835914	48490.9/8	21	790	
L46	glyceraldehyde 3-phosphate dehydrogenase A subunit	gi|62318887	MDP0000527995 c	17730.1/5.35	6	210	
L47	NADP-dependent glyceraldehyde-3-phosphate dehydrogenase;	gi|3913711	MDP0000152497	53654.6/6.76	10	375	
L53	alcohol dehydrogenase	gi|307135978	MDP0000239956	40657.6/8.53	8	216	
L59	chloroplast sedoheptulose-1,7-bisphosphatase	gi|118175929	MDP0000244771	42824.9/6.06	9	377	
L83	PREDICTED: triosephosphate isomerase	gi|225449541	MDP0000152242	27396.2/6.34	6	266	
L86	PREDICTED: pyruvate dehydrogenase E1 component subunit beta	gi|357159289	MDP0000146411	40081.6/5.47	5	407	
Binding proteins							
L68	ATP synthase, subunit B	gi|7578491	MDP0000565338	53365/5.57	15	752	
L69	ATP synthase subunit B	gi|91983091	MDP0000565338	51812.1/5.27	21	703	
L71	ATP synthase, subunit B	gi|7578491	MDP0000565338	53365/5.57	19	791	
L72	putative ATP synthase subunit B	gi|56784991	MDP0000565338	45936.9/5.33	17	971	
L73	PREDICTED: ATP synthase subunit B, mitochondrial-like	gi|225424142	MDP0000565338	59699.2/5.84	18	760	
L77	ATP synthase, subunit B	gi|7578491	MDP0000565338	53365/5.57	19	632	
L78	ATP synthase CF1 alpha subunit	gi|346683279	MDP000092905	55443.9/5.09	21	1100	
L79	ATP synthase subunit B	gi|91983091	MDP0000565338	51812.1/5.27	24	923	
Antioxidant related proteins							
L25	Probable aldo-keto reductase 2	gi|378548276	MDP0000228499	38436.6/6.17	6	341	
L37	aldo/keto reductase AKR	gi|62526573	MDP0000228499	38026.5/6.38	10	483	
Defense and stress related proteins							
L13	class III endo-chitinase	gi|33347391	MDP0000280265	17774.6/4.12	1	207	
L16	heat shock protein 70	gi|30962610	MDP0000322220	47584/5.1	9	106	
L17	beta-1,3-glucanase	gi|399137110	MDP0000570395	37551.2/5.07	4	258	
L18	thioredoxin peroxidase	gi|21912927	MDP000020081	30103.4/8.2	5	328	
L22	thaumatine-like protein	gi|20149274	MDP0000552328	22948.5/4.6	7	317	
L28	ascorbate peroxidase	gi|145581388	MDP0000199034	27711.8/5.53	6	414	
L29	ascorbate peroxidase	gi|319993039	MDP0000194474	12064.3/8.72	3	73	
L30	early-responsive to dehydration 2	gi|383100964	MDP0000322220	69263.1/5.19	11	149	
L84	quinone oxidoreductase [Gymnadenia conopsea]	gi|89276317	MDP0000393227	21631.1/6.06	2	159	
L89	phi class glutathione S-transferase protein	gi|329130898	MDP0000266097	23996.3/5.97	3	90	
L90	IgE-binding protein MnSOD	gi|10862818	MDP0000187714	22957.7/6.06	6	397	
Unknown proteins							
L20	uncharacterized protein LOC100805310	gi|359806638	MDP0000875642	27959.1/5.29	9	353	
L31	PREDICTED: uncharacterized protein At2g37660, chloroplastic	gi|225440390	MDP0000176370	27681.5/5.85	2	150	
L32	predicted protein	gi|224087915	MDP0000641719	27403.9/5.74	9	464	
L38	PREDICTED: clavaminate synthase-like protein At3g21360	gi|225442460	MDP0000406399	36588.7/6.1	4	80	
L57	predicted protein	gi|224138316	MDP0000148186	45396.1/6.11	14	534	

*In relative protein content graph, from left to right were : Control group 3 days post-treatment, : Inoculated group 3 days post-treatment, : Control group 6 days post-treatment, : Inoculated group, 6 days post-treatment respectively.

### Functional classification and subcellular localization of pathogenically induced proteins

**Figure 3:**
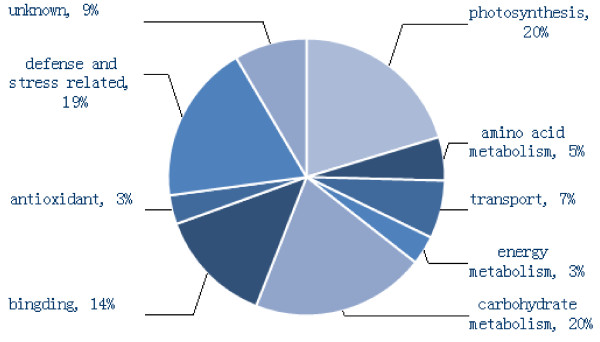
**Functional categories and percentage in each functional category of identified protein spots differentially expressed in the isolated ‘Qinguan’ leaves inoculated with *M. coronaria.***

The fifty-nine successfully identified protein spots were grouped according to the biological process using the GO annotation in the *Green Plant* database (http://www.geneontology.org/). The identified proteins fall into 9 functional categories including photosynthesis (twelve spots, 19%), amino acid metabolism (3 spots, 5%), transport (4 spots, 7%), energy metabolism (2 spots, 3%), carbohydrate metabolism (twelve spots, 20%), binding (8 spots, 14%), antioxidant (2 spots, 3%), defense and stress (eleven spots, 19%) and unknown (5 spots, 8%). The most important categories related to infectivity included defense and stress related proteins, accounting for 19% of the total, and antioxidant related proteins which accounted for 3% (Table 1, Figure 3).

**Figure 4:**
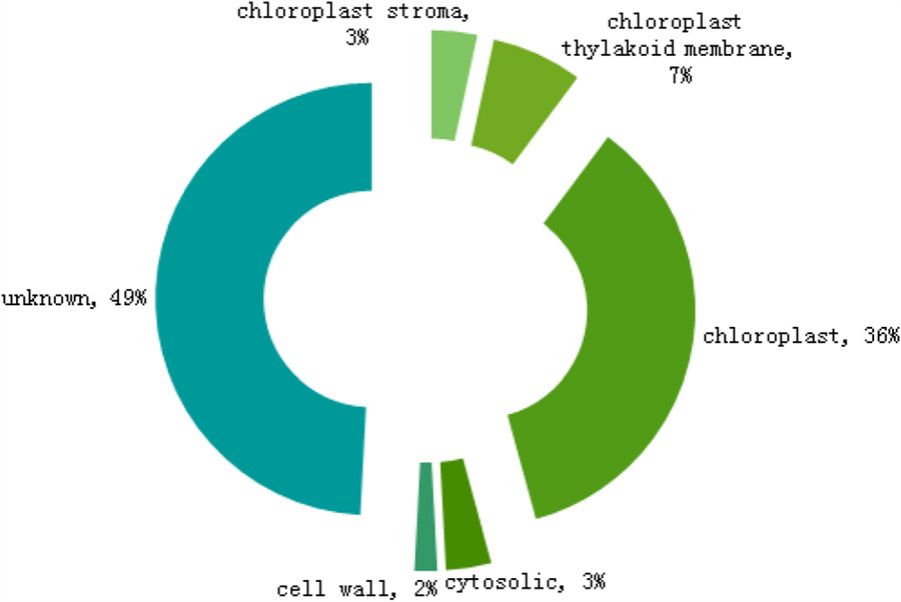
**The subcellular location classification and percentage of protein spots differentially expressed in the isolated ‘Qinguan’ leaves inoculated with *M. coronaria.***

Subcellular location analysis provides important information about the physiological function of identified proteins [17]. The GO annotations have been widely used to predict the locations of proteins because the two are strongly correlated [18,19]. Some of the identified proteins (L8, L50, L51, L82, L85, L95, L58, L27, L83, L68, L69, L71, L72, L73, L77, L78, L79, L56, L99, L23 and L59) were localized to chloroplasts, and others to stroma (L45, L49), thylakoid membrane (L15, L19, L61 and L87), cytosol (L36, L41), and the cell wall (L89). Localization of all other proteins could not be determined (Figure 4).

### Protein abundance change in response to *M. coronaria* inoculation in apple leaves

After inoculation, fifty-nine protein spots were identified successfully, classified into 3 groups: proteins that showed increased in protein abundance only at 3 day after *M. coronaria* inoculation compared to the control (Group A); proteins that showed increased in protein abundance versus control at 6 day timepoint in response to inoculation (Group B); and proteins that showed increased in protein abundance in the inoculation group at both 3 day and 6 day timepoints (Group C).

The thirty-three protein spots were identified in Group A, including 8 related to photosynthesis (Rubisco, L8, L45, L49, L51, L82, L85, L95; oxygen-evolving enhancer protein 2, L50), 2 related to amino acid metabolism (L43, L54), 1 related to transport (transketolase, L56), 2 related to energy metabolism (L23, L24), eleven related to carbohydrate metabolism (L27, L33, L36, L41, L42, L46, L47, L53, L59, L83, L86), 4 related to protein binding (ATP synthase, L71, L72, L73, L77), 3 related to defense and stress response (heat shock protein 70, L16, ascorbate peroxidase, L28, L29, early-responsive to dehydration 2, L30) and 2 with unknown function (L31, L57).

Eight protein spots were identified in Group B, including 2 proteins related to photosynthesis (chlorophyll a-b binding protein, L15, L19), 1 protein related to carbohydrate metabolism (glyceraldehyde-3-phosphate dehydrogenase A, L40), 1 protein related to protein binding (ATP synthase CF1 alpha subunit, L78), 1 protein related to antioxidant function (probable aldo-keto reductase 2, L25), 2 proteins related to defense and stress (beta-1,3-glucanase, L17, quinine oxidoreductase, L84) and 1 unknown protein (L32).

There are twelve protein spots in group C, including 2 proteins related to photosynthesis (chlorophyll a-b binding protein, L61, L87), 1 protein related to amino acid metabolism (putative plastidic glutamine synthetase, L58), 1 protein related to transport (alanine aminotransferase 3, L81), 2 proteins related to binding (ATP synthase, B subunit, L 68, L79), 1 protein related to antioxidant function (aldo/keto reductase AKR, L37), 4 proteins related to defense and stress (class III endo-chitinase, L13, thaumatine-like protein, L22, lgE-binding protein MnSOD, L90, phi class glutathione S-transferase protein, L89), and one unknown protein (L38).

**Figure 5:**
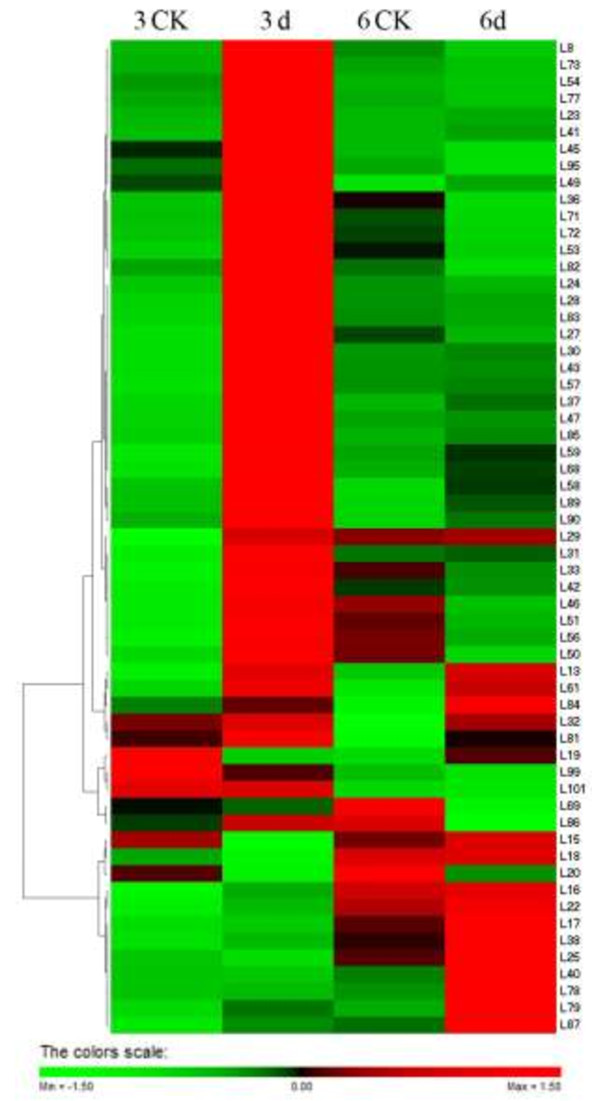
**Clustering analysis of 2-DE gel data.** Data from 59 differentially expressed protein spots (the spot IDs such as L* have been detected by MALDI-TOF-TOF/MS) that showed an at least 1.5-fold change in the relative volume between the inoculated and non-inoculated leaves were subjected to two-way hierarchical cluster analysis, performed with PermutMatrix. Pearson’s distance and Ward’s algorithm were used. Each colored cell represents the average of the relative spot volumes, according to the color scale depicted at the bottom of the figure. 3CK-Noninoculated leaf after 3 day (treated with sterile water alone). 3d-Inoculated with *M. coronaria* leaf after 3 day. 6CK- Non-inoculated leaf after 6 day (treated with sterile water alone). 6d-Inoculated with *M. coronaria* leaf after 6 day.

Cluster analysis of 2-D electrophoretic data of fifty-nine identified protein spots was performed with PermuMatrix (Figure 5). Among thirty-three protein spots increased in protein abundance at 3 day treatment and eight protein spots at 6 day after inoculation. These results imply that the different protein abundance can occur at a slow or fast onset in response to pathogenesis.

### Transcription level change of genes encoding identified proteins in response to *M. coronaria* inoculation in apple leaves

**Figure 6:**
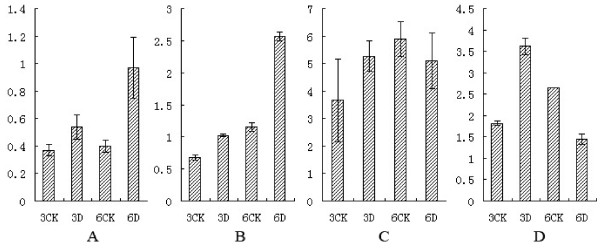
**qRT-PCR identification of four differential expression genes between the inoculated with *M. coronaria *(3D and 6D) and non-inoculated (3CK and 6CK). A**: Relative expression of class III endo-chitinase (Spot L13), **B**: beta-1, 3-glucanase (Spot L17), **C**: thaumatine-like protein (Spot L22), **D**: ascorbate peroxidase (Spot L28). The vertical axis represents the relative levels of mRNA. From left to right: 3CK-Noninoculated leaf after 3 day (treated with sterile water alone). 3D-Inoculated with *M. coronaria* leaf after 3 day. 6CK- Noninoculated leaf after 6 day (treated with sterile water alone). 6D-Inoculated with *M. coronaria* leaf after 6 day.

In order to determine the relationship between the proteome data and transcriptome ones, four genes encoding identified proteins correlated with disease resistance were selected for qRT-PCR verification: class III endo-chitinase (L13), beta-1, 3-glucanase (L17), thaumatine-like protein (L22), and ascorbate peroxidase (L28). Primer pairs for each gene are shown in Table 2. The analysis showed that transcription levels of each gene changed over time in response to *M.coronaria* (Figure 6).

**Table 2:** **Gene-specific primer pairs used for qRT-PCR analysis of four identified genes in ‘Qinguan’ leaves inoculated with** ***M. coronaria***

**Spot no.**	**Gene name**	**Accession no.**	**Primer sequence (5’-3’)**
L13	Class III endo-chitinase	gi|33347391	Forward: GCACTCAACGGACACAAC
			Reverse: GTAGAACTGAACCCAAACG
L17	Beta-1, 3-glucanase	gi|399137110	Forward: AGTCGTATCGGAGAGTGGTTG
			Reverse: TGAGTTGGTACTTGGGTTGTTT
L22	Thaumatine-like protein	gi|20149274	Forward: ATGGTCTGGTCGCTTCTG
			Reverse: CCGTCAACAAGGCTAACA
L28	Ascorbate peroxidase	gi|145581388	Forward: AAGGTGCCACAAGGAGCG
			Reverse: AGAGGGCGGAAGACAGGG

The transcription levels of class III endo-chitinase (L13) and beta-1, 3-glucanase (L17) are approximately consistent with the proteomics level. However, the mNA abundance of thaumatine-like protein (L22) and ascorbate peroxidase (L28) are not. The transcription levels of thaumatine-like protein (L22) had no obvious change. Ascorbate peroxidase (L28) increased the gene abundance at 3 day compared to control, but decreased at 6 day compared to control.

## Discussion

To our knowledge, this is the first comparative proteomic study of apple leaf response induced by *M. coronaria*. In this study, we obtained a overview of the altered change in protein abundance in apple leaves responding to *M. coronaria* infection. We identified 59 protein spots involved in photosynthesis, amino acid metabolism, transport, energy metabolism, carbohydrate metabolism, binding, antioxidant, defense and stress. The results of the proteome analysis are discussed below and mainly focus on the groups of the defense and stress and antioxidant related proteins.

### Photosynthesis related proteins

Twenty percent of the protein spots identified successfully were involved in photosynthesis. The induction of some photosynthetic proteins during the interaction between apple leaves and *M. coronaria* may implicate light-sensing mechanisms in the induction of plant disease defense signaling. A series of proteins related to photosynthesis were altered suggesting the dynamic influence of pathogen on host photosynthetic machinery [20,21]. Some plants show a decrease in the activity of some Calvin cycle-related proteins, e.g., RuBisCO, following pathogen infection [22,23]. The up-regulation of the RuBisCO at 3 day can be explained by the rapid defense response to the fungus. It is probable that pathogens cause a gradual decline in the rate of photosynthesis in infected areas of the plants as the disease progresses [23]. An up-accumulation of related proteins could be part of a defense strategy. At 6 day, the down-regulation of protein activity indicated that infection with *M. coronaria* possible inhibited the rate and extent of photosynthetic processes. This down-regulation of photosynthesis during pathogen infection may be caused by the large accumulation of hexose sugars, which leads to feedback inhibition of the expression of some photosynthetic genes [24,25]. It is conform to the report by Xianping Fang et al [16].

### Defense and stress related proteins and antioxidant related proteins

Twenty-two percent of the protein spots identified in this study were involved in defense and stress related and antioxidant related proteins. Plants have developed sophisticated mechanisms to protect themselves against pathogen infections. Their immunity can be triggered by pattern-recognition receptors (PRRs) that act via pathogen-associated molecular patterns (PAMP) and effectors. This recognition leads to defense responses, such as oxidative bursts, the induction of pathogenesis-related proteins, and the deposition of callose to strengthen the cell wall at sites of infection [26].

Hypersensitive Response (HR) is the most distinguishing hallmark of resistance and is characterized by rapid localized plant cell death at the site of infection [27,28]. The HR generates a physical barrier composed of dead cells and limits the availability of nutrients to the pathogen which can further restrict its spread. Other defense related responses often accompany HR, such as oxidative burst, pathogenesis related proteins [29].

Oxidative burst, which involves the production of reactive oxygen species (ROS), is a ubiquitous response of plants to pathogen attack following successful pathogen recognition. ROS has been proposed as orchestrating the establishment of these defence responses [30]. ROS-scavenging systems have an important role in regulating the amount of ROS that is generated [31]. Fungal elicitation generates a rapid oxidative burst; the host plant curtails the propagation of toxic products in order to localize the cell death [32]. In agreement with previous study, the antioxidative enzymes, for example, ascorbate peroxidase [33], quinine oxidoreductase [34], lgE-binding protein MnSOD [34], Phi class glutathione S-transferase protein [35] and aldo/keto reductase AKR, have been shown to be up-accumulated in response to *M. coronina* infection. Phi class glutathione S-transferase protein plays a key role in cellular detoxification by conjugating glutathione (GSH) to a wide variety of substrates [36]. Plant Phi class glutathione S-transferase protein can also act as a GSH peroxidase [37,38]. This protein can protect cells from oxygen toxicity and suppressing apoptosis [39]. These antioxidative enzymes were involved in the removal of peroxides, oxidation of toxic reductant, biosynthesis and degradation of lignin, suberization, auxin catabolism and responses to environmental stresses such as wounding, pathogenic attack and oxidative stress [40,41].

In the recent years, many aspects referred to Systemic Acquired Resistance (SAR) have been elucidated. The pathway leading to SAR involves three steps, pathogen recognition, signal relay and induction of genes, which facilitate synthesis of protective molecules. Induction of PR-1, 2, 5 and 8 is characteristic of SAR in several herbaceous plants. But very little molecular evidence for SAR in wood perennials has been reported. Jean M Bonasera et al identified four genes as candidates for involvement in the response of apple to attack by *E. amylovora* based on their similarity to genes documented as involved in SAR in other plants. Three of the four apple genes, PR-2, PR-5 and PR-8, but not PR-1 is up-regulated in response to inoculation with the pathogen *E. amylovora*. In our results, PR-2 (beta-1, 3-glucanase), PR-5 (Thaumatine-like protein) and PR-8 (Class III endo-chitinase) increased in protein abundance in response to inoculation with the pathogen *M. coronaria*, not PR-1, which conform to the result of PR gene described by Jean M Bonasera et al and Van Loon et al. [42,43].

Chitinase gene and beta-1, 3-glucanses gene were demonstrated to be multigene family among apple species. Different members in the gene family existed expression difference by inoculation induction. The induced expression of class III endo-chitinase (L13, gi|33347391, MDP0000280265) and beta-1, 3-glucanses (L 17, gi|399137110,MDP0000570395) were shown in this study.

It was previously possible to detect the proteins chitinase and beta-1, 3-glucanses in the apoplast fluid of the *M. domestica*[44]. Chitinase and beta-1, 3-glucanase could protect plants against fungal infection in two ways. First, these proteins can directly weaken and decompose the fungal cell walls [45]. Second, oligosaccharide elicitors, released through those digested walls, can induce a consequent chain of defense reactions [46]. Activities by chitinase and beta-1, 3-glucanase can be significantly induced by infection, as shown in several pathosystems [47,48]. Additionally, the activities of those are higher during an incompatible interaction than in one that is compatible [49]. Thaumatine-like protein (L22), a class 5-PR protein (PR-5), has previously exhibited a number of anti-fungal properties [50]. Moreover, the transgenic expression of thaumatine-like protein engendered anti-fungal activity through the inhibition of mycelial growth [51,52]. These proteins identified in this work were generally consistent with previous studies on the transcriptional and proteomics response of disease. In agreement with the work of Venisse et al. Chitinase and beta-1, 3 glucanase were up-regulated in response to *E. amylovora* challenge [53]. In agreement with the work of Xianping Fang et al. [16], beta-1, 3-glucanase was up-regulated in response to *C. fragariae* of strawberry leaves.

The constitutive expression of the PR proteins, especially in the apoplast of the *M.domestic*, and possibly also in other parts of the leaf, is most probably responsible for strengthening the cell wall, as well as for rapid degradation of *M. coronaria* mycelium and for the failure of the fungus to complete its life cycle in the apoplast. It is well documented in the literature that plant defence against pathogens involves an oxidative burst and that the reactive oxygen species not only damage the pathogen but also the plant itself [54].

### The other proteins

In plants, pathogenic infections often induce some common physiological alterations, for example amino acid, energy and carbohydrate metabolism, transport, binding and et al. In our study, enzymes involved these functions were found to be differentially regulated in response to *M. coronaria* infection. Ferredoxin-NADP reductase (L23) is a key enzyme of electrons. Ferredoxin-NADP reductase transfers electrons between the one-electron carrier ferredoxin and the two-electron carrier NADP (H) at the end of the electron transport chain. This reaction provides the NADPH necessary for CO2 assimilation in plants. Ferredoxin-NADP reductase also participates in other relevant processes as the electron cyclic flow around the photosystem I and in the control of the NADPH/NADP + homeostasis of stressed chloroplasts [55]. After SCMV infection, ferredoxin-NADP reducase was up-accumulated at 3 day, while recovered at 6day. The result indicates that the ability of energy metabolism might increase at 3 day, however recovered at 6 day. Glyceraldehyde-3-phosphate dehydrogenase (L42), the key enzyme of carbohydrate metabolism, increased in protein abundance. The expression patterns consistent with those reported previously [56]. We speculated that *M. coronaria* infection might result in the signal recognition particle (SRP) to synthesize more glucose, fructose and betaine. The synthesis of these energy substances can maintain intracellular homeostasis.

### Unknown proteins

In addition to the positively identified proteins in this study, a number of differentially expressed proteins could not be associated with an existing biological function. These candidate proteins will require further study in the future.

### Correlation between pathogenically induced gene transcription level abundance and protein abundance

**Figure 7:**
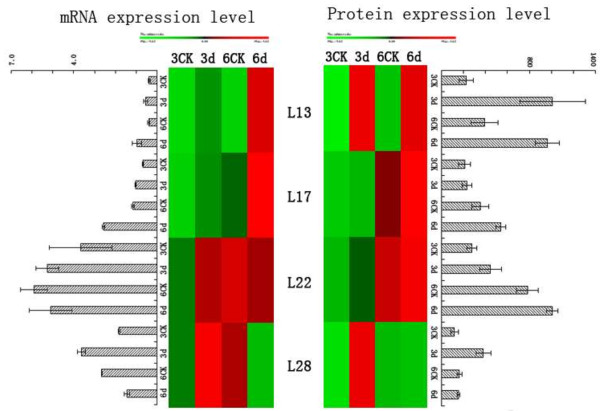
**Comparison of the mRNA and protein expression levels for four identified genes in ‘Qinguan’ leaves inoculated with *M. coronaria.*** qRT-PCR was performed using gene-specific primers (Table 2). To achieve a more intuitive understanding of the correlation between mRNA and protein expression, hierarchical cluster analysis was performed with PermutMatrix. L13-class III endo-chitinase (Spot L13), L17- beta-1, 3-glucanase (Spot L17), L22- thaumatine-like protein (Spot L22), L28-ascorbate peroxidase (Spot L28). 3CK-Non-inoculated leaf after 3 day (treated with sterile water alone). 3d-Inoculated with *M. coronaria* leaf after 3 day. 6CK- Non-inoculated leaf after 6 day (treated with sterile water alone). 6d-Inoculated with *M. coronaria* leaf after 6 day.

In order to determine the correlation between levels of protein abundance and their corresponding mRNA level, the relative abundance of four genes including class III endo-chitinase (L13), beta-1, 3-glucanase (L17), thaumatine-like protein (L22), and ascorbate peroxidase (L28) was assessed at 3 and 6 days post-inoculation. Hierarchical clustering was also performed to achieve a more intuitive overview of the correlation between pathogen induced gene abundance and protein abundance (Figure 7). Variation in mRNA levels of class III endo-chitinase (L13) and beta-1, 3-glucanase (L17) exhibited a good relation with the proteomic level. Transcription levels of thaumatine-like protein (L22) had no obvious difference. Conversely, mRNA abundance of ascorbate peroxidase (L28) was only increased at 3 day post-inoculatio and decrease at 6 day. Despite the fact that protein abundance has no change at 6 day. The inconsistency between transcription and translation level does not account for their independence, and perhaps their major roles are regulatory effects including transcriptiona regulation, differential processing of RNA transcript and differential translation of mRNA [57]. The inconsistency between transcriptional and translational levels is influenced by many factors, and thus the further verification is required to elucidate its mechanisms.

## Conclusion

In a conclusion, different proteins in response to *Marssonina coronaria* were identified by proteomic analysis. Among of these proteins, there are some PR proteins, for example class III endo-chitinase, beta-1,3 glucanase and thaumatine-like protein, and some antioxidant related proteins including aldo/keto reductase AKR, ascorbate peroxidase and phi class glutathione S-transferase protein that were associated with disease resistance. The transcription levels of class III endo-chitinase (L13) and beta-1, 3-glucanase (L17) have a good relation with the data of the proteomics level, however, the mRNA abundance of thaumatine-like protein (L22) and ascorbate peroxidase (L28) are not. To elucidate the resistant mechanism, the data in the present study will promote us to investigate further the expression regulation of these target proteins.

## Methods

### Plant material and *M. coronaria* inoculation

Uniform and healthy mature ‘Qinguan’ apple leaves were obtained from apple repository of Northwest A & F University. After harvest, the leaves were immediately surface-sterilized with 8% sodium hypochlorite for 10 min. The petiole was rinsed with sterile water, air-dried, and wrapped with sterile cotton. Treatment with sterile water was used as a control versus *M. coronaria* inoculation, performed by pipetting 20 conidial suspension spots (10^6^ conidia ml^−1,^ 20 μl per infection spot) on the upper leaf surface. The leaves were cultured in an incubator at 25°C with a relative humidity of 95-100%. Leaves from treatment and control were collected at 3 and 6 days post-inoculation, immediately frozen in liquid nitrogen and stored at -70°C until further use.

A monosperse culture of *Marssonina* blotch-derived *Diplocarpon mail* was collected from Northwest A & F University. Single spore isolation was performed according to procedures outlined by Lee et al. [6].

### Total protein extraction and 2-D gel electrophoresis of apple leaves

Three independent replicates of each apple leaf sample were crushed in a pre-cooled mortar with liquid nitrogen until a fine powder was formed. Total protein content was extracted with TCA-phenol, as described previously [58]. Precipitated protein was re-suspended in sample buffer containing 7 M Urea (Merck), 2 M Thiourea (Sigma Aldrich), 2% (w/v) CHAPS (Sigma Aldrich), 2% (v/v) DTT (Sigma Aldrich) and Bromophenol blue traces (Sigma Aldrich). Protein concentration was determined via Bradford assay (BioRad), diluted to a final concentration of 3 ug/ul, and stored at -20°C.

Protein samples (total 1000 ug,350 ul) were loaded onto 17 cm pH 4-7 IPG strips with active rehydration loading buffer for 1 hour in sample buffer (described previously) with the addition of 0.2% (v/v) IPG buffer (pH 4-7). Isoelectric focusing (IEF) was performed on a Bio-Rad IEF system at 25°C using the following protocol: S1 linear 250 V 30 min, S2 rapid 500 V 30 min, S3 rapid 1000 V 1 h, S4 linear 10,000 V 4 h, S5 rapid 10,000 V 60 kVh, S6 rapid 500 V 24 h. Subsequently the IPG strips were equilibrated by gentle shaking for 15 min in equilibration buffer I [6 M urea, 2% (w/v) SDS, 0.374 M Tris-HCl (pH 8.8), 20% (w/v) glycerol, 2% (w/v) DTT] followed by 15 min in equilibration buffer II (2.5% iodoacetamide instead of DTT). Each strip was then transferred onto vertical slab 12% SDS-polyacrylamide gels. Electrophoresis was run at 15°C for 30 min at 80 V followed by 100 V for 10 h. The gels were stained with colloidal Coomassie Brilliant Blue G-250 [59], washed three times with Milli-Q water for 5 mins and fastened in fixative solution [40% (v/v) ethanol, 10% (v/v) acetic acid and 10% (v/v) carbinol] for 1 h. After a second set of Milli-Q water washes, the gel was stained with colloidal Coomassie Brilliant Blue [0.1% (w/v) CBB G-250, 10% (w/v) ammonium sulfate, 1.2% (v/v) phosphoric acid and 20% (v/v) ethanol] overnight. Finally, background staining was removed with destaining solution [10% (v/v) ethanol, 10% (v/v) acetic acid].

### Gel image acquisition and cluster analysis

Gel images were acquired using a Powerlook2100XL optical density scanner and imported into PDQuest 8.0.1 image software (Bio-Rad, Hercules CA, USA) for analysis. A total of 36 gels, resulting from three technical replicates for each biological replicate, were analyzed. A 1.5-fold change in protein expression between inoculated and control states was deemed to be statistically significant. PermutMatrix was used to conduct cluster analysis for different treatments, with parameters set as following: Dissimilarity: Pearson’s distance, Hierarchical: Ward’s Minimmu Variance Method, Used dataset: Normalize Rows (z-score). After analysis by PDQuest image software, differential protein spots were excised from the preparative gels and stored in 2 ml Eppendorf tubes.

### Protein identification by MALDI-TOF-TOF/MS Analysis

Gel fragments were destained with 300 μl 100 mM NH_4_HCO_3_ and 30% acrylonitrile (ACN). After removed the destaining buffer using 100% ACN, the gel pieces were lyophilized. The dry gel fragments were rehydrated in 5 μl 2.5-10 ng/μl trypsin (Promega, Madison, WI, USA) for approximately 20 h. After removal of hydrolysate, the remaining peptides were extracted in 100 μl 60% ACN by sonication. Extracts were pooled together and lyophilized. The resulting lyophilized tryptic peptides were kept for mass spectrometric analysis.

MS spectrum analysis was performed with a 4800 Plus MALDI TOF/TOF™ Analyzer (Applied Biosystems, USA). Analysis was completed on behalf of The Biochemistry and Cell Biology Shanghai Institute for Biological Sciences, Chinese Academy of Sciences.

### Database search and protein identification

MS spectral data obtained was analyzed using GPS Explore software, and the results of each sample were integrated into one file. The results were searched against the NCBI nr database using MASCOT software (Matrix Science, London, U.K.). Protein content was determined using PD-Quest software.

### Total RNA isolation and Quantitative Real Time-PCR

Total RNA was isolated from inoculated and control leaves by the CTAB method described by Ksenija et al with modifications [60]. RNase-free DNase I (TaKaRa, Dalian, China) was used to eliminate genomic DNA according to the manufacturer’s instructions. The integrity of RNA was checked by electrophoresis using a 1% agarose gel. RNA was reverse-transcribed using the PrimeScript RT reagent Kit With gDNA Eraser (TaKaRa, Dalian, China) according to manufacturer instructions.

Protein candidates highly correlated with disease resistance were selected for qRT-PCR examination. The *M. domestica* housekeeping gene actin-2 was used as an endogenous reference for relative quantification. The following actin-2 primers were used: forward primer 5‘-CGATGGCCAAGTCATCACAAT-3’, reverse primer 5‘-GACCCACCACTGAGCACGATG-3’ [61]. The qRT-PCR was set to cycling parameters of 95°C for 2 min followed by 40 cycles of 95°C for 30s, 58°C for 30s, and 72°C for 30s.

## Competing interests

The authors declare that they have no competing interests and declare no conflict of interest.

## Authors’ contributions

MML: participated on study design and all experimental procedures as well as the manuscript draft. JHX and JZ: participated on experimental procedures. ZHQ: participated on experiment execution. JKZ: performed the study design and coordinated the manuscript draft. All authors read and approved the final manuscript.
